# Epinephrine infusion as an adjuvant treatment for breakthrough reactions during desensitization to methotrexate^☆^

**DOI:** 10.1016/j.waojou.2024.100965

**Published:** 2024-09-18

**Authors:** Carla Toledo-Salinas, David Alejandro Mendoza-Hernandez, Paul J. Turner

**Affiliations:** a Grupo Médico Pediátrico, Mexico City, Mexico; b Coordinating Commission of National Institutes of Health and Advanced Specialty Hospitals, Mexico City, Mexico; c National Institute of Pediatrics, Mexico City, Mexico; d National Heart Lung Institute, Imperial College London, London, UK

To the editor:

Desensitization protocols can induce temporary tolerance to a drug which has previously caused an allergic response, and thus facilitate appropriate and timely medical management.^1^ Rapid desensitization protocols have proven safe and cost-effective; in the context of cancer treatment, desensitization can allow patients to remain on first-line therapy, which can improve morbidity and survival.^2^ Breakthrough allergic reactions are frequent during desensitization, but tend to remit during subsequent procedures.^3^ We describe a patient who continued to experience recurrent breakthrough anaphylaxis events during methotrexate desensitization. Use of low dose epinephrine infusions allowed desensitization to proceed successfully.

A 13-year-old female was diagnosed with tibial osteoblastic osteosarcoma. Her treatment regime included methotrexate (MTX). While receiving the first dose of MTX, she developed acute-onset erythema, fever and mild rigors, nausea and vomiting, consistent with a “mixed type” (IgE- and cytokine-mediated) phenotype. Basophil activation test was positive to MTX (25 mg/ml) (69% activation gated as CD63^+^CD123^+^HLA-DR^-^).

We undertook a 13-step rapid desensitization protocol for MTX (see Table 1) during each of the patient's nine procedures. During the first procedure, she developed acute-onset generalized erythema, rigors and tachycardia during step 12. Subsequently the patient was given an additional dose of antihistamine (chloropyramine) and mefenamic acid prior to step 10. Unfortunately, this did not prevent progression to anaphylaxis during subsequent infusions (see Table 1). Discussion with the oncology team reinforced their preference to use MTX, as it is the cornerstone of treatment for osteosarcoma, in combination with salvation surgery. We therefore decided to co-administer a low dose intravenous epinephrine infusion (0.1 mcg/kg/minute) during subsequent desensitization, as an adjunct treatment. This allowed the patient to complete MTX administration without symptoms (although she did develop erythema 15 min after stopping the epinephrine infusion after the sixth administration). Thereafter, the epinephrine infusion was commenced prior to desensitization, which resulted in the absence of any allergic features.

**Table 1:**
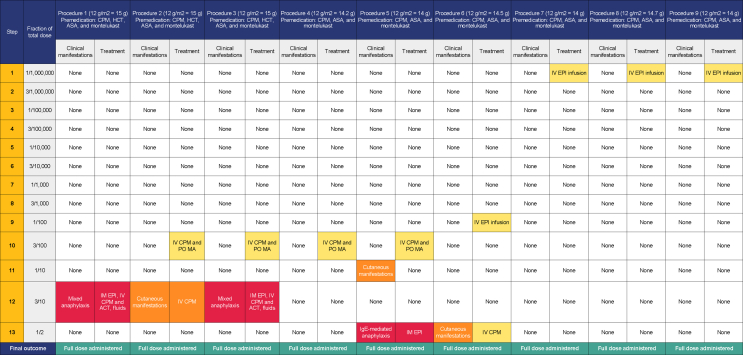
Rapid drug desensitization protocol. *ACT, acetaminophen (paracetamol); ASA, acetylsalicylic acid; CPM, chloropyramine; EPI, epinephrine; HCT, hydrocortisone; MA, mefenamic acid*

MTX is widely used to treat hematologic and non-hematologic malignancies; hypersensitivity reactions are infrequent.^4^ We opted for a 13-step desensitization protocol since this may reduce the risk of adverse reactions in patients with hypersensitivity to monoclonal antibodies and chemotherapeutic agents.^5^ Breakthrough reactions are not infrequent,^6^ and tend to remit during subsequent procedures.^3^^,^^6^ Our patient continued to experience allergic symptoms despite desensitization with appropriate premedication.

Intramuscular (IM) epinephrine is the first-line treatment of anaphylaxis; however, many guidelines include the use of continuous, low dose intravenous epinephrine infusion when initial treatment with IM epinephrine fails.^7^^,^^8^ Under specialist expert supervision, low dose epinephrine infusions are safe;^9^^,^^10^ indeed, their use has become more widespread in the management of shock associated with COVID-19 in both high and low-income countries.^11^ We achieved successful administration of MTX using adjunct epinephrine infusion at the lower dose recommended by international guidelines for the treatment of refractory anaphylaxis. Adjunct low-dose epinephrine infusion may be a useful option in low-resource settings where alternative options (such as anti-IgE therapy) are not feasible, so long as appropriate cardiovascular monitoring and personnel with the relevant expertise are available.

Sincerely,

CarlaToledo-Salinas, MD

David Alejandro Mendoza-Hernandez, MD

Paul J.Turner, MD, PhD

## Abbreviations

IM, Intramuscular; MTX, methotrexate.

## Consent to publish

We confirm that we have obtained consent to publish case information from the participant and their legal guardian for this Letter to the Editor. We thank the patient and her family for providing consent.
